# Supplementary material for: Hepatitis B virus cccDNA is formed through distinct repair processes of each strand
Source: Nat Commun. 2021 Mar 11;12:1591. doi: 10.1038/s41467-021-21850-9 (PMC7952586; doi:10.1038/s41467-021-21850-9)
Supplement: Supplementary file 1 — Supplementary Information [file 41467_2021_21850_MOESM1_ESM.pdf]

## Supplementary information

for

# Hepatitis B virus cccDNA is formed through distinct repair processes of each strand

Lei Wei[1] and Alexander Ploss[1,*].

[1]Department of Molecular Biology, Lewis Thomas Laboratory, Princeton University, Washington Road, Princeton, NJ, 08544, USA

*Correspondence should be address to A.P. (aploss@princeton.edu)

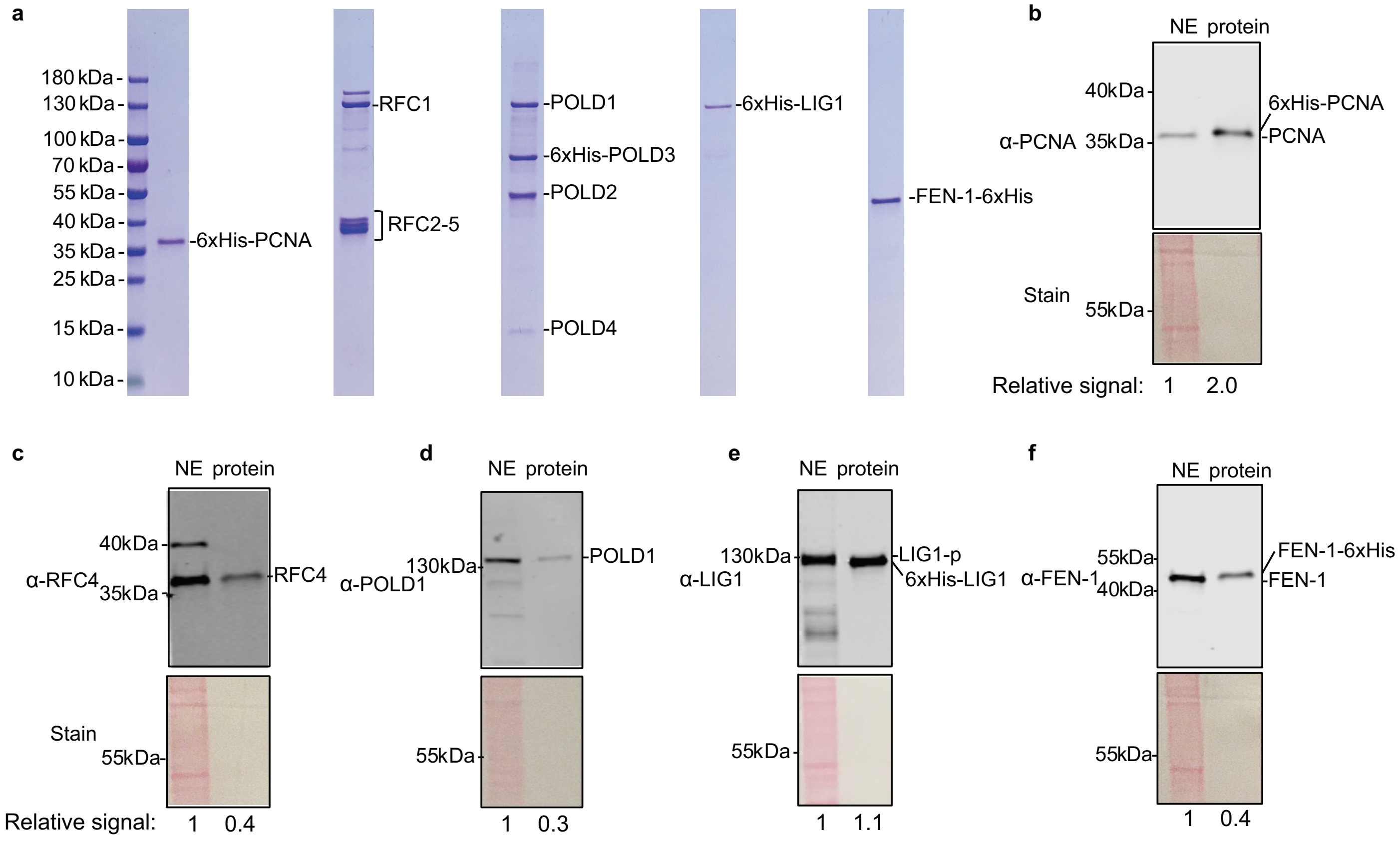


**Supplementary Fig. 1. Analyses of purified recombinant proteins used in the cccDNA formation assays**. (**a**) All five purified human protein factors involved in cccDNA formation assay were analyzed by SDS–PAGE with Coomassie blue staining. (**b-f**) The relative amounts of each human protein factors in both human hepatoma cell line hNTCP-HepG2 nuclear extracts (14 mg $ml^{-1}$) and in vitro assays with purified proteins were analyzed by western blotting. Relative signal was calculated by normalizing the signal of a specific band of a protein to that of the nuclear extract (NE). Note that LIG1 in nuclear extract is phosphorylated and has a slower mobility, as previously shown[20]. NE, nuclear extracts; protein, purified protein system; stain, Ponceau S stain. All experiments were repeated twice with similar results. Source data are provided as a Source Data file.

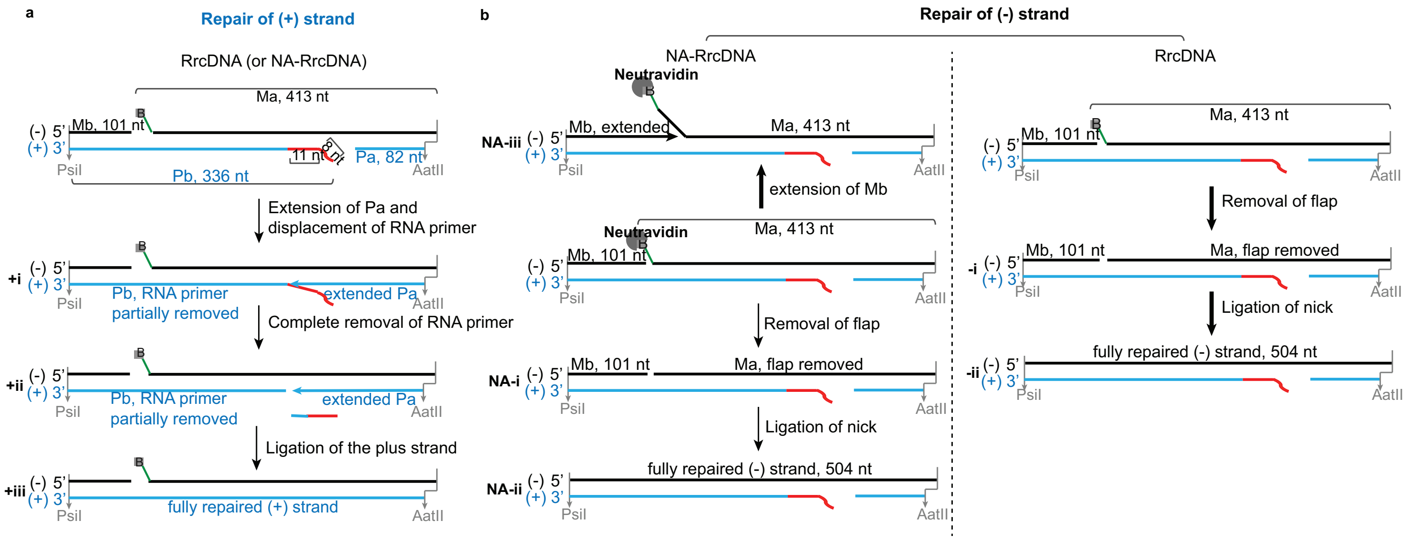


**Supplementary Fig. 2. Schematic of the HBV rcDNA repair process in the purified human factor system.** (**a**) Model of the repair process of the plus-strand. The complete removal of the RNA primer requires the extension of Pa to reach the 5' terminus of Pb and to displace the RNA primer to form a flap structure (+i). The RNA flap will then be completely removed by FEN-1 (+ii). After complete RNA removal, the two fragments on the plus-strand are ligated and the plus-strand is fully repaired (+iii). (**b**) Model of the repair process of the minus-strand. The repair efficiency of the minus-strand is influenced by the presence of a protein adduct. Left, when the protein adduct is present, around 10-20% of the DNA flap will be removed by FEN-1, leaving only one nick on the minus-strand (NA-i). This nick is then sealed, leading to a fully repaired minus-strand (NA-ii). The majority of the flap with the protein adduct will not be removed by FEN-1. In this situation, the 3' end of the minus-strand ssDNA will be elongated by PCNA-POLδ and displaces the 5' end of the minus-strand, generating long DNA flaps (NA-iii). Right, when the protein adduct is absent, over 90% of the flap on the minus-strand will be removed by FEN-1 within 1 min, leaving a single nick (-i). This nick will then be ligated to complete the repair of the minus-strand (-ii).

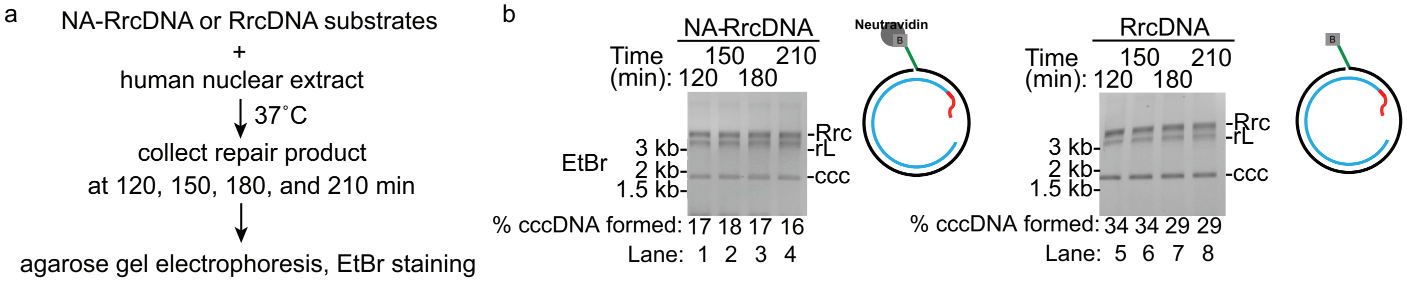


**Supplementary Fig. 3. Extended incubation of recombinant rcDNA substrates with human nuclear extracts beyond cccDNA formation plateau does not further increase cccDNA formation efficiency.** (**a**) Schematic for examining the effect of extended incubation time in cccDNA formation. (**b**) Time course assay (120-210 min) showing the kinetics of cccDNA formation from both NeutrAvidin (NA)-RrcDNA (lanes 1−4) and RrcDNA (lanes 5−6) as described in (a). The percentage of cccDNA formed was calculated as in Fig. 2c and indicated in row '% cccDNA formed' above the lane numbers. All experiments were repeated twice with similar results. Rrc, RrcDNA; rL, recombinant linear RrcDNA, ccc, cccDNA. Source data are provided as a Source Data file.

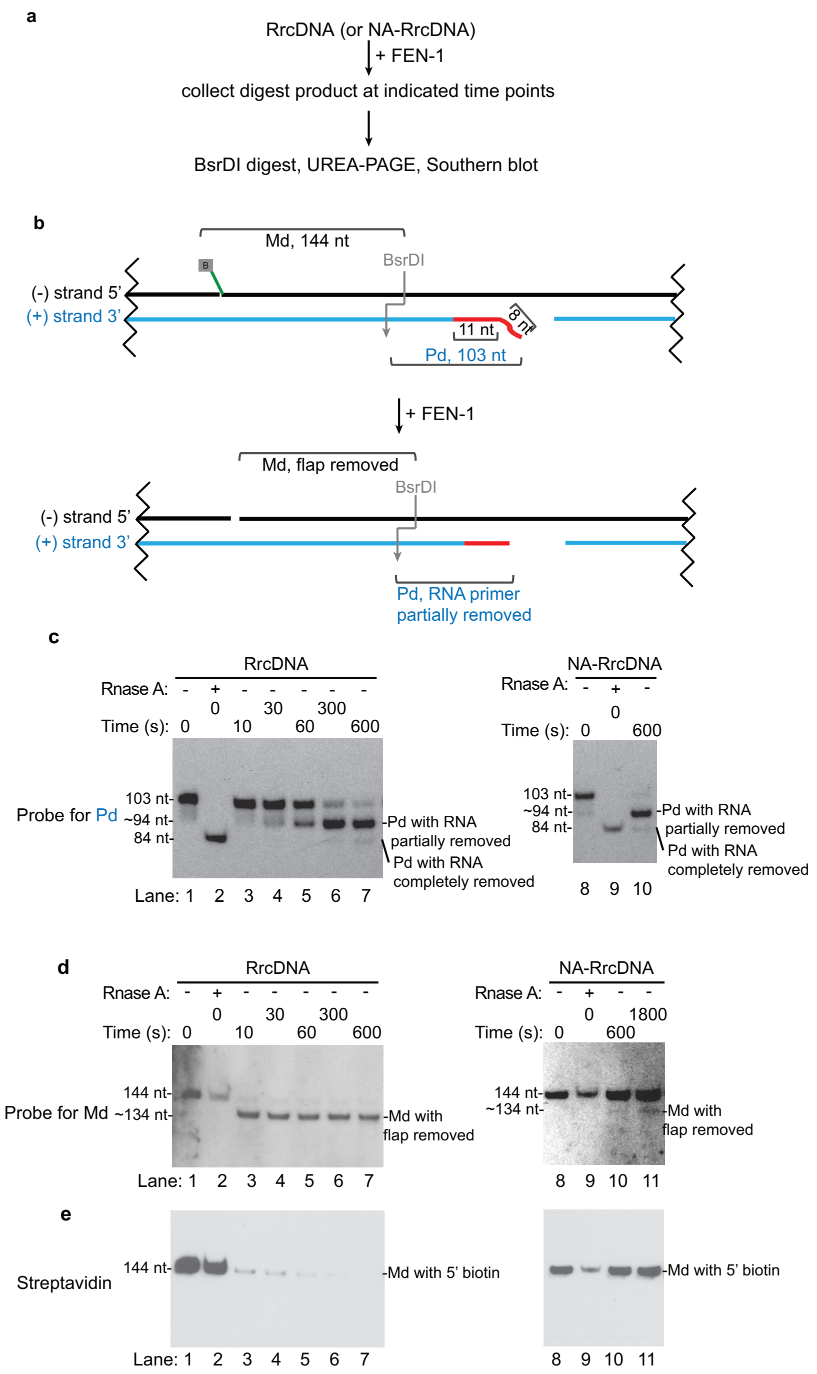


**Supplementary Fig. 4. Function of FEN-1 in removal of the DNA flap on the minus-strand and the RNA primer on the plus-strand.** (**a**) Schematic for examining the effect of FEN-1 on removal of the DNA flap on the minus-strand and the RNA primer on the plus-strand. (**b**) Schematic describing the effects of FEN-1 alone in the removal of DNA and RNA flaps on RrcDNA. Green line, biotinylated flap; red line, RNA primer. Note that in the 19 nt RNA primer, 8 nt do not pair with the minus-strand and form a RNA flap. BsrDI cleavage (indicated by gray arrows) was used to examine the removal of the flaps. (**c**) As described in (a-b), the removal of the RNA flap from the plus-strand Pd fragment by FEN-1 was examined by Southern blot using probes specific for the plus-strand. ~, size estimation, since there is a short range for FEN-1 cleavage sites. (**d-e**) The removal of DNA flap from the minus-strand Md fragment by FEN-1 was examined by Southern blot (d) and Streptavidin blot (e). Note that the presence of protein adduct drastically inhibits the removal of DNA flap (compare lanes 2-3 and lanes 8-11). All experiments were repeated twice with similar results. Source data are provided as a Source Data file.

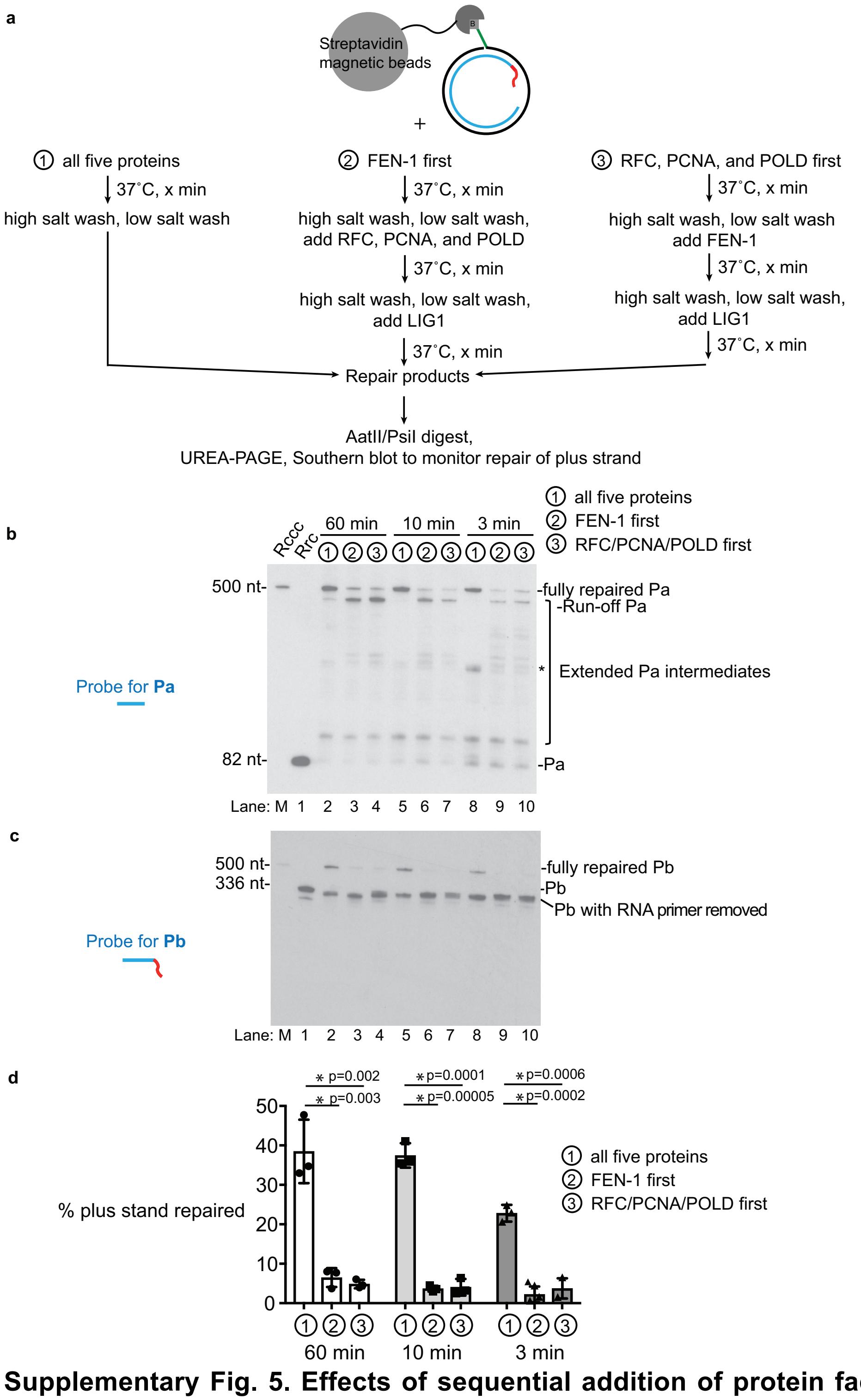


**Supplementary Fig. 5. Effects of sequential addition of protein factors on the plus strand repair of recombinant HBV rcDNA**. (**a**) Schematic of sequential addition of protein factors in cccDNA formation assays. RrcDNA is immobilized on magnetic streptavidin beads and protein factors could be added, washed off, allowing sequential incubation of factors with the substrates. (**b-c**) Repair of plus-strand fragments Pa (b) and Pb (c), were monitored by Southern blot. (**d**) The percentage of plus strand repaired was calculated from (c) as described in Fig. 2i, and plotted. The experiments were repeated three times and all three individual measurements were shown in the plot. Bar values represent average of three measurements, and error bars indicate s.d. Statistical analyses of the cccDNA formation efficiencies between indicated pairs were performed by two-stage step-up t-test method from Graphpad Prism. Source data are provided as a Source Data file.

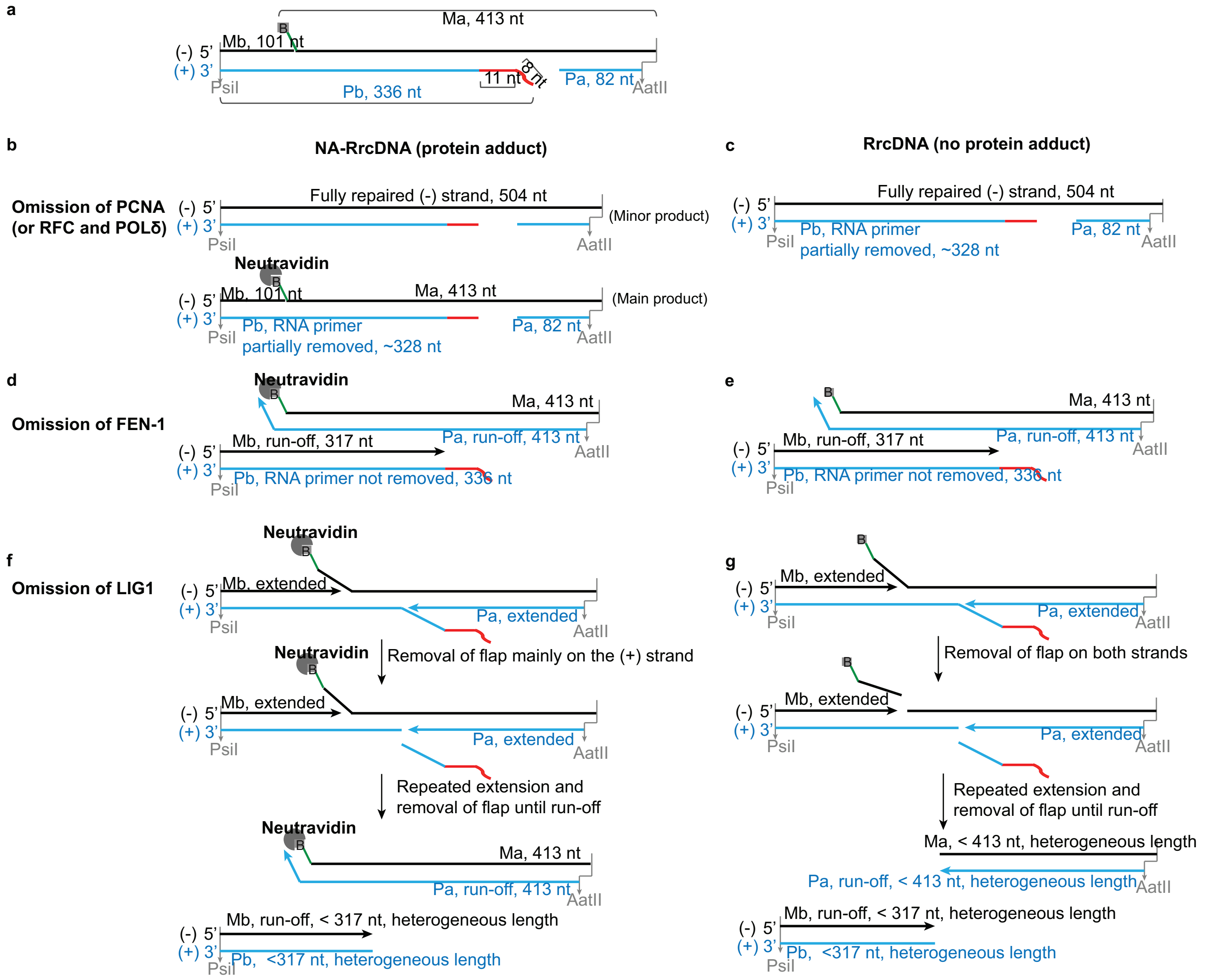


**Supplementary Fig. 6. Schematic of repair intermediates when individual repair factors are omitted.** (**a**) A simplified schematic depicting the four fragments in unrepaired RrcDNA digested by AatII/PsiI. (**b-c**) Repair intermediates of NA-RrcDNA (b) or RrcDNA (c) when PCNA (or RFC and POLδ) is omitted. Note that in (b), only 10-20% of minus-strands are completely repaired (minor product), and most minus strand lesions are not processed (major product), due to the presence of a protein adduct which interferes with the removal of DNA flap, while virtually all minus-strands are repaired in (c). The RNA primer on the plus-strand is partially removed by FEN-1, since Pa is not extended by POLδ to completely displace the RNA primer for its removal. (**d-e**) Repair intermediates of NA-RrcDNA (d) or RrcDNA (e) when FEN-1 is omitted. Without FEN-1, the DNA flap and RNA primer are not processed, and the 3' end of the plus- and minus-strands will be engaged and elongated by PCNA-POLδ until template run-off, linearizing the RrcDNA substrates. (**f-g**) Repair intermediates of NA-RrcDNA (f) or RrcDNA (g) when LIG1 is omitted. When the protein adduct is present on the minus-strand (f), the 5' end flap on the minus-strand is largely unprocessed. The 3' end of the minus-strand (Mb) is elongated until run-off. Similarly, the 3' end of the plus-strand (Pa) is extended and displaces Pb until run-off. However, FEN-1 degrades Pb gradually as it gets displaced by Pa, therefore the final intermediate is similar to those when FEN-1 is omitted, but shorter in the Mb and Pb repair intermediates. When the protein adduct is absent on the minus-strand (g), the process of the plus-strand is the same as in (f), with one difference in the processing of minus-strand, as the majority of the flap can be removed, Ma is degraded by FEN-1 as Mb displaces Ma. Therefore, the final intermediate is shorter than those in (f).

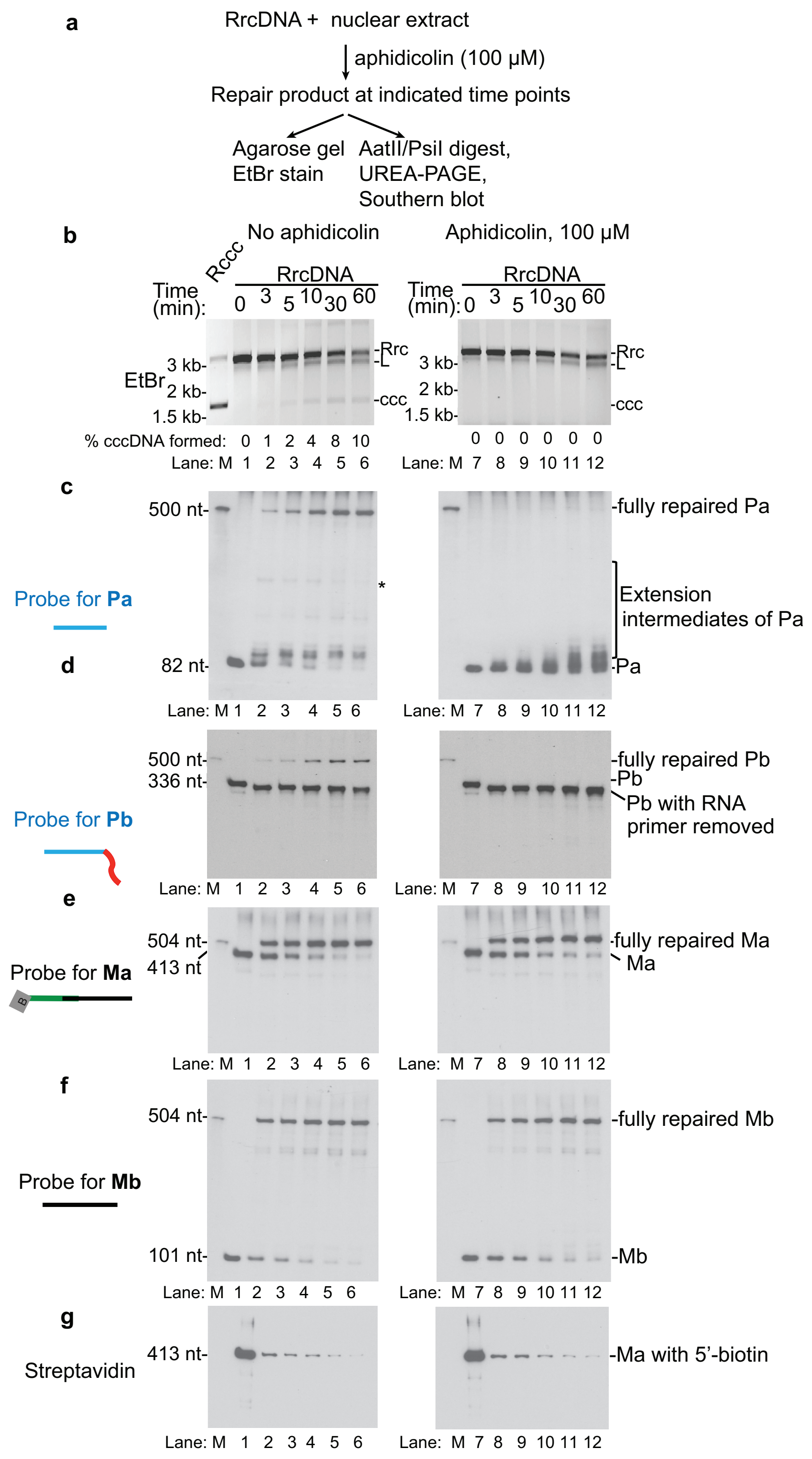


**Supplementary Fig. 7. Aphidicolin specifically inhibits the repair of the rcDNA plus-strand in human nuclear extracts.** (**a**) Schematic of time course experiments to test the effects of aphidicolin on repair of HBV RrcDNA plus and minus strands repair. (**b**) cccDNA formation of the RrcDNA substrate in human nuclear extracts under treatments of mock (1% DMSO, lanes 1-6) or aphidicolin (100 μM in 1% DMSO, lanes 7-12) was detected by EtBr-stained agarose gel. (**c-g**) Repair of plus-strand fragments Pa, Pb, and minus-strand fragments Ma, Mb was monitored by Southern blot (c-f) or streptavidin blot (g). All experiments were repeated twice with similar results. Rrc, RrcDNA; rL, recombinant linear RrcDNA, ccc, cccDNA. Source data are provided as a Source Data file.

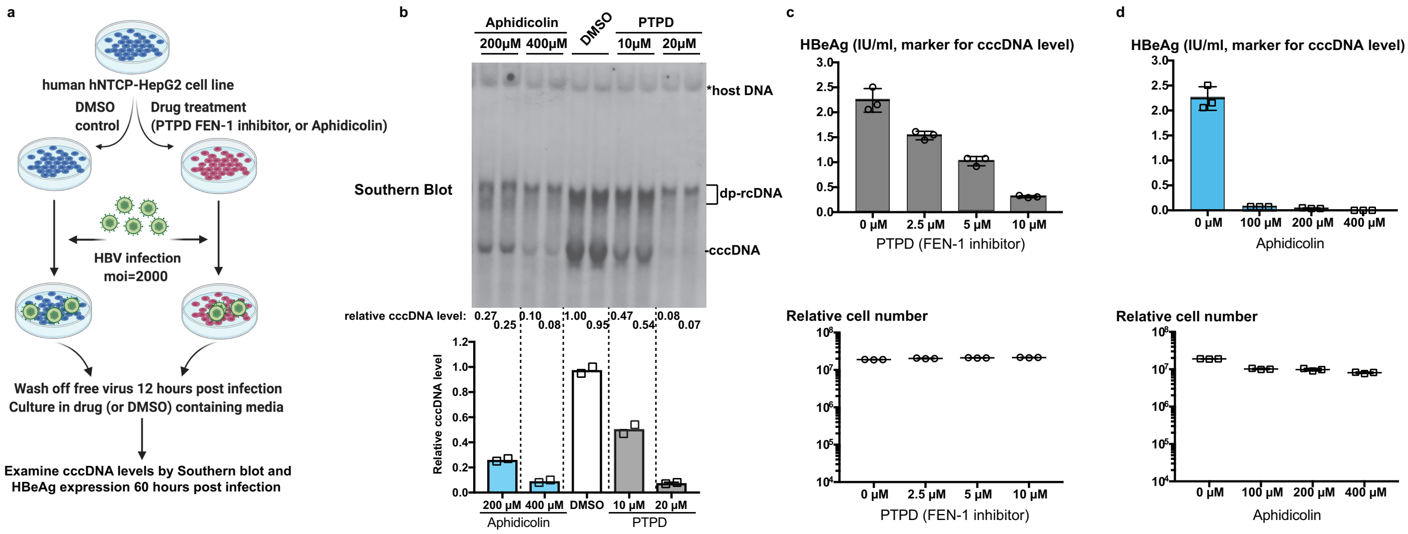


**Supplementary Fig. 8. FEN-1 and POLδ inhibitors potently suppressed cccDNA levels in cells infected with HBV.** (**a**) Schematic of examining the effects of FEN-1 and POLδ inhibitors (PTPD and aphidicolin) in cccDNA formation in cells infected with HBV. (**b**) Upper panel, PTPD and aphdicolin inhibit cccDNA levels in cells infected with HBV in a dose dependent manner. * Indicates host DNA in Hirt extraction samples that either contains integrated HBV and/or is non-specifically detected by HBV probes. These host DNA bands also indicate equal loading of samples. Lower panel, relative cccDNA signals from upper panel were quantified and plotted. The signal of cccDNA level from DMSO treated cells was set to 1. The bar values indicate the average of two measurements. (**c**) FEN-1 inhibitor PTPD efficiently reduces HBeAg levels (by HBeAg ELISA) in cells infected with HBV in a dose dependent manner (upper panel), without detectable toxicity (lower panel, by CellTiter-Glo 2.0, Promega). (**d**) Same as (c), except that aphidcolin was tested. Note that the slightly reduced cell number (lower panel) was due to aphicolin inhibits cell proliferation, and no cell death was observed at time of harvest. Independent experiments in (b) were repeated twice. Independent experiments in (c-d) were repeated thrice. The bar values in (c-d) indicate the average of three measurements and the error bars are the s.d. Source data are provided as a Source Data file.

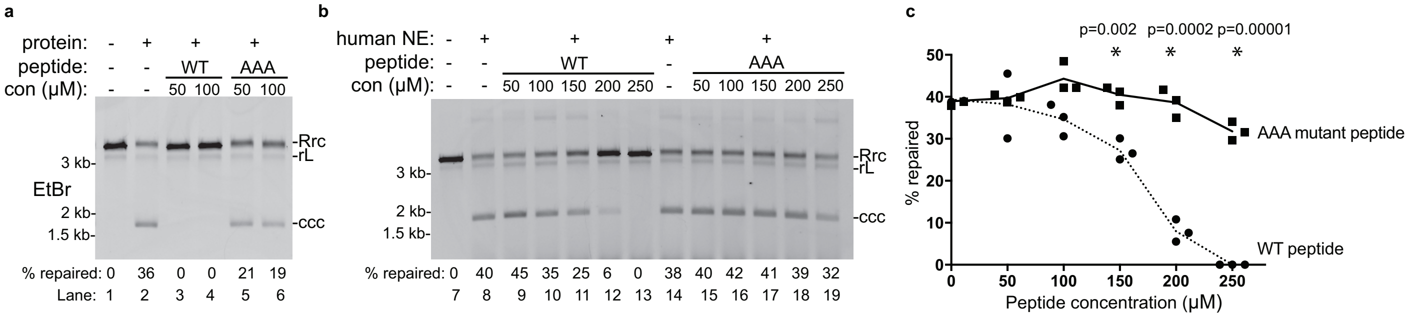


**Supplementary Fig. 9. Cyclin-dependent kinase inhibitor p21 peptide abrogates cccDNA formation in purified protein and human nuclear extract systems.** (**a-b**) WT p21 peptide, but not the mutant AAA peptide, blocks PCNA-POLδ interaction, diminishes cccDNA formation in the purified human protein system (a) and nuclear extract system (b) in a dose-dependent manner. % repaired, the percentage of total RrcDNA that is repaired to form cccDNA, was calculated by the intensity of the cccDNA band divided by the sum intensities of RrcDNA, rL and cccDNA bands. Absolute values are shown above each lane number. (**c**) Efficiency of cccDNA formation from (b) was calculated and plotted. Rrc, recombinant rcDNA; rL, linear recombinant rcDNA; ccc, cccDNA. Experiments in (a) were repeated twice with similar results. Experiments in (b) were repeated 3 times, and all three measurements are plotted in (c). The lines connect the average value of 3 measurements at indicated concentrations. Statistical analyses between cccDNA formation efficiencies at indicated concentrations are performed by two-stage step-up t-test method from Graphpad Prism, and statistically significant p values are indicated by '*'. Source data are provided as a Source Data file.

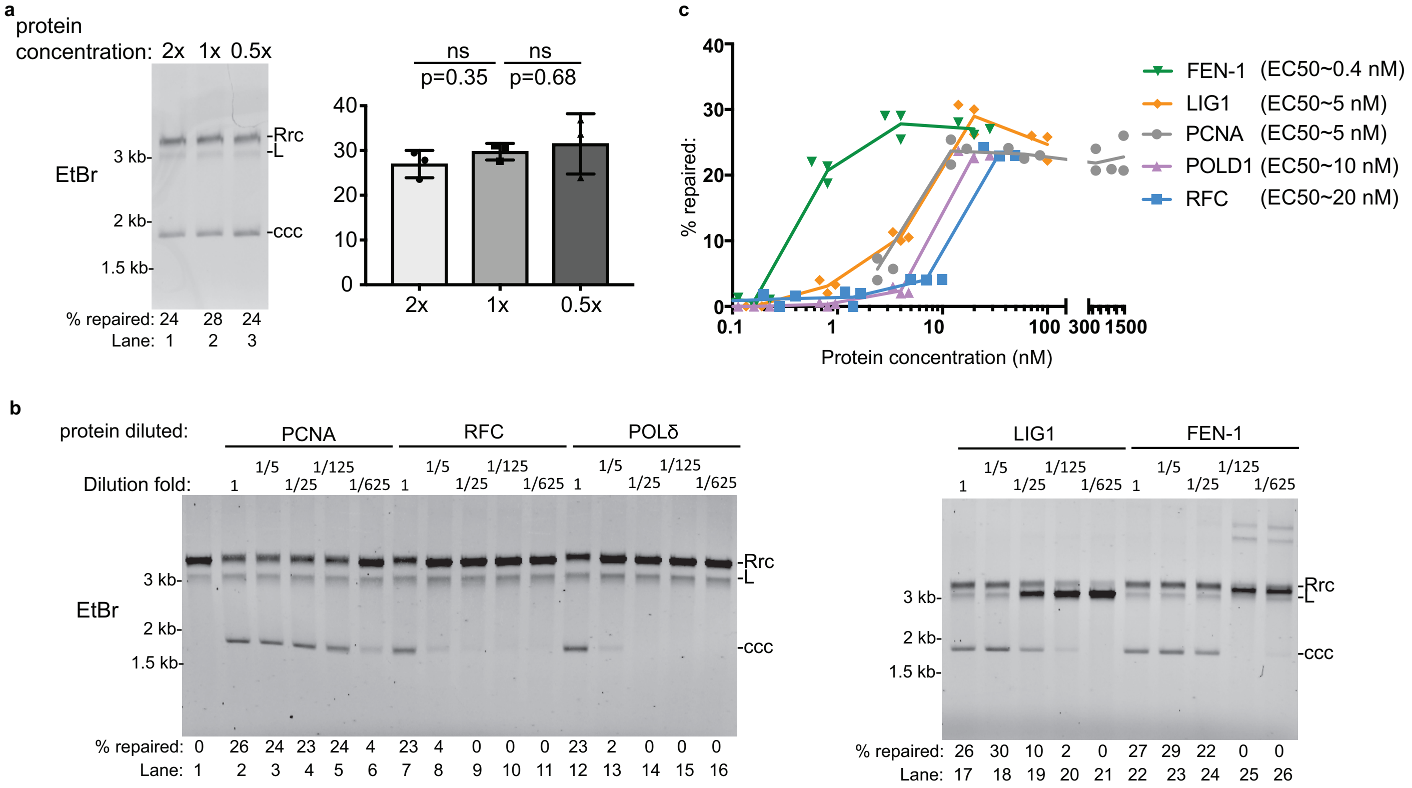


**Supplementary Fig. 10. Concentration requirements of the five human repair factors in cccDNA formation.** (**a**) The cccDNA formation efficiency with five human repair factors (1x, 1.5 μM PCNA, 35 nM RFC, 20 nM POLδ, 100 nM LIG1 and 20 nM FEN-1) reached maximal level. 2x and 0.5x indicates that the concentrations of all five proteins are two-fold or half, respectively, of the amounts in 1x. % repaired, the percentage of total RrcDNA that is repaired to form cccDNA as in Supplementary Fig. 5c. The bar values indicate the average of three measurements and the error bars are the s.d. (**b**) cccDNA formation efficiency was tested when the concentration of the indicated protein was serially diluted while the concentrations of all other four proteins remained constant. (**c**) Efficiency of cccDNA formation from (b) was calculated and plotted. All experiments were repeated 3 times, and each individual measurement is plotted. The lines in (c) connect the average values of 3 measurements at indicated concentrations. Rrc, RrcDNA; rL, recombinant linear RrcDNA, ccc, cccDNA. Source data are provided as a Source Data file.

Supplementary Table 1. Oligos used in this study.

| | |
|---|---|
| ssDNA probe for Pa fragment | ACGGCAGACGGAGAAGGGGACGAGAGAGTCCCAAGCGACCCCGAGA AGGGTCGTCCGCAGGATTCAGCGCCGACGGGACGTAAACAAAGG |
| ssDNA probe for Pb fragment | TAAGGGTCGATGTCCATGCCCCAAAGCCACCCAAGGCACAGCTTGGA GGCTTGAACAGTAGGACATGAACAAGAGATGATTAGGCAGAGG |
| ssDNA probe for Ma fragment | CCTTTGTTTACGTCCCGTCGGCGCTGAATCCTGCGGACGACCCTTCTC GGGGTCGCTTGGGACTCTCTCGTCCCCTTCTCCGTCTGCCGT |
| ssDNA probe for Mb fragment | CCTCTGCCTAATCATCTCTTGTTCATGTCCTACTGTTCAAGCCTCCAAG CTGTGCCTTGGGTGGCTTTGGGGCATGGACATCGACCCTTA |
| ssDNA probe for Pd fragment (related to supplementary Fig. 4c) | GACATTGCAGAGAGTCCAAGAGTCCTCTTATGTAAGACCTTGGGCAAC ATTCGGTGGGCGTTCACGGTGGTCTCCATGCGACGTGCAGAG |
| ssDNA probe for Md fragment (related to supplementary Fig. 4d) | AACGACCGACCTTGAGGCATACTTCAAAGACTGTTTGTTTAAAGACTG GGAGGAGTTGGGGGAGGAGATTAGATTAAAGGTCTTTGTACT |
