# Supplementary material for: Hepatitis B virus cccDNA is formed through distinct repair processes of each strand
Source: Nat Commun. 2021 Mar 11;12:1591. doi: 10.1038/s41467-021-21850-9 (PMC7952586; doi:10.1038/s41467-021-21850-9)
Supplement: Supplementary file 3 — Reporting Summary [file 41467_2021_21850_MOESM3_ESM.pdf]

nature research

Corresponding author(s): Alexander Ploss

Last updated by author(s): 2021/01/31

Reporting Summary

Nature Research wishes to improve the reproducibility of the work that we publish. This form provides structure for consistency and transparency in reporting. For further information on Nature Research policies, see our Editorial Policies and the Editorial Policy Checklist.

Statistics

For all statistical analyses, confirm that the following items are present in the figure legend, table legend, main text, or Methods section.

n/a	Confirmed	
☐	☒	The exact sample size (n) for each experimental group/condition, given as a discrete number and unit of measurement
☐	☒	A statement on whether measurements were taken from distinct samples or whether the same sample was measured repeatedly
☐	☒	The statistical test(s) used AND whether they are one- or two-sided<br>*Only common tests should be described solely by name; describe more complex techniques in the Methods section.*
☒	☐	A description of all covariates tested
☐	☒	A description of any assumptions or corrections, such as tests of normality and adjustment for multiple comparisons
☐	☒	A full description of the statistical parameters including central tendency (e.g. means) or other basic estimates (e.g. regression coefficient) AND variation (e.g. standard deviation) or associated estimates of uncertainty (e.g. confidence intervals)
☐	☒	For null hypothesis testing, the test statistic (e.g. F, t, r) with confidence intervals, effect sizes, degrees of freedom and P value noted<br>*Give P values as exact values whenever suitable.*
☒	☐	For Bayesian analysis, information on the choice of priors and Markov chain Monte Carlo settings
☒	☐	For hierarchical and complex designs, identification of the appropriate level for tests and full reporting of outcomes
☒	☐	Estimates of effect sizes (e.g. Cohen's d, Pearson's r), indicating how they were calculated

Our web collection on statistics for biologists contains articles on many of the points above.

Software and code

Policy information about availability of computer code

Data collection	EtBr stained gels were scanned with Typhoon FLA 9500; Signals from Southern blots were visualized on autoradiography film, and then scanned with an Epson scanner at 300 dpi resolution
Data analysis	The signals from EtBr stained gels and Southern blots were all quantified with ImageJ version 1.52a, and plotted with Graphpad Prism 7.0d Software

For manuscripts utilizing custom algorithms or software that are central to the research but not yet described in published literature, software must be made available to editors and reviewers. We strongly encourage code deposition in a community repository (e.g. GitHub). See the Nature Research guidelines for submitting code & software for further information.

Data

Policy information about availability of data

All manuscripts must include a data availability statement. This statement should provide the following information, where applicable:

- Accession codes, unique identifiers, or web links for publicly available datasets
- A list of figures that have associated raw data
- A description of any restrictions on data availability

All data are available in the manuscript or the Extended Data File.

Field-specific reporting

Please select the one below that is the best fit for your research. If you are not sure, read the appropriate sections before making your selection.

☒ Life sciences ☐ Behavioural & social sciences ☐ Ecological, evolutionary & environmental sciences

For a reference copy of the document with all sections, see nature.com/documents/nr-reporting-summary-flat.pdf

Life sciences study design

All studies must disclose on these points even when the disclosure is negative.

Sample size	For all biochemical experiments, sample sizes (n=2 or 3) were chosen according to the standard of previous publications in the field [e.g. Cannavo, E. et al. Regulation of the MLH1-MLH3 endonuclease in meiosis. Nature. 586, 618-622 (2020); and Douglas, M. E., Ali, F. A., Costa, A. & Diffley, J. F. X. The mechanism of eukaryotic CMG helicase activation. Nature. 555, 265-268 (2018)].
Data exclusions	No data exclusions
Replication	All data shown were repeated with at least two independent experiments with similar results. The criteria used to confirm reproducibility are that all independent experiments reach the same conclusions.
Randomization	There was no randomization for biochemical experiments, because randomization is not relevant to the experiments performed in this study, similar to a previous publication by Cannavo, E. et al. Regulation of the MLH1-MLH3 endonuclease in meiosis. Nature. 586, 618-622 (2020).
Blinding	There was no blinding for biochemical experiments, since blinding is not relevant to the experiments performed (similar to the publication listed above.

Reporting for specific materials, systems and methods

We require information from authors about some types of materials, experimental systems and methods used in many studies. Here, indicate whether each material, system or method listed is relevant to your study. If you are not sure if a list item applies to your research, read the appropriate section before selecting a response.

Materials & experimental systems

n/a	Involved in the study	
☐	☒	Antibodies
☐	☒	Eukaryotic cell lines
☒	☐	Palaeontology and archaeology
☒	☐	Animals and other organisms
☒	☐	Human research participants
☒	☐	Clinical data
☒	☐	Dual use research of concern

Methods

n/a	Involved in the study	
☒	☐	ChIP-seq
☒	☐	Flow cytometry
☒	☐	MRI-based neuroimaging

Antibodies

Antibodies used	Primary antibodies used were anti-PCNA (for western blotting, validated by recombinant hPCNA, 1:500 dilution, clone PC10, sc-56, lot# E2418, Santa Cruz Biotechnology, Dallas, TX), anti-PCNA (for immune-depletion, validated by recombinant hPCNA, clone F2, sc-25280, lot #H1417, Santa Cruz Biotechnology, Dallas, TX), anti-POLD1 (validated by recombinant hPOLD1, 1:1000 dilution, rabbit polyclonal, 15646-1-AP, Proteintech, Rosemont, IL), anti-FEN-1 (validated by recombinant hFEN-1, 1:1500 dilution, rabbit polyclonal, A300-256A, Bethyl Laboratories, Montgomery, TX), anti-LIG1 (validated by recombinant hLIG1, 1:1000 dilution, clone 10H5, GTX70141, lot#809703999, GeneTex, Irvine, CA), and anti-RFC4 (validated by recombinant hRFC4, 1:1000 dilution, clone 1320, GTX70285, lot#14130, GeneTex, Irvine, CA).
Validation	We validated antibodies against PCNA, LIG1, FEN-1, POLD1, and RFC4 by western blot using purified recombinant proteins, as shown in Extended Data Figure 1.

Eukaryotic cell lines

Policy information about cell lines

Cell line source(s)	HepG2 cells was purchased from American Tissue Culture Collection, ATCC® Number: HB-8065™, Manassas, VA; hNTCP-expressing HepG2 cells clone 3B10 was previously described in (Winer, et al. 2017).

Authentication	hNTCP-expressing HepG2 3B10 cell line was authenticated by its susceptibility to HBV infection. 3B10 cell line is a derivative of HepG2 cell line.
Mycoplasma contamination	All tested free of Mycoplasma contamination.
Commonly misidentified lines (See ICLAC register)	No commonly misidentified cell lines were used in the study.
